# Supplementary material for: Network analysis shows decreased ipsilesional structural connectivity in glioma patients
Source: Commun Biol. 2022 Mar 23;5:258. doi: 10.1038/s42003-022-03190-6 (PMC8943189; doi:10.1038/s42003-022-03190-6)
Supplement: Supplementary file 2 — Supplementary Information [file 42003_2022_3190_MOESM2_ESM.pdf]

Supplementary Table 1. iFOD2 significant edges after TFNBS.

Subgroups	Edges	FWE adjusted P values
Whole	POP-PaCG	.0206
Whole	PTR-CMFG	.0006
Whole	PTR-PaCG	.001
Whole	PCG-PTR	.0206
Whole	RMFG-CMFG	.0144
Whole	SFG-IPG	.0144
Whole	SFG-MTG	.0144
Whole	SFG-POP	.0046
Whole	SFG-PTR	.0446
Whole	SFG-PrCG	.0024
Whole	SMG-CMFG	.002
Whole	SMG-PTR	.0206
Whole	SMG-SFG	.001
Whole	TTG-SFG	.0144
Whole	TH-SFG	.0206
Whole	CA-MOFG	.0446
Whole	CA-PaCG	.006
Whole	PU-MOFG	.0206
Whole	PU-PaCG	.006
Whole	PU-PoCG	.0144
Whole	PU-PCG	.0084
Whole	PU-PrCG	.0446
Whole	PU-SFG	.0084
Whole	PA-PaCG	.0144
Whole	PA-PrCG	.0144
Whole	PA-SFG	.0024
Whole	CeC-PrCG	.006
Whole	CeC-SFG	.0144
Whole	BS-CACG	.0446
Whole	BS-PaCG	.0294
Pecentral	POP-PaCG	.0062
Pecentral	PTR-CMFG	.0226
Pecentral	PTR-PaCG	.0008
Pecentral	PoCG-CMFG	.0226
Pecentral	PCG-POP	.0062
Pecentral	PrCG-CMFG	.009
Pecentral	RMFG-CMFG	.0182
Pecentral	SFG-CMFG	.0056
Pecentral	SFG-PrCG	.0026
Pecentral	SPG-CMFG	.0226
Pecentral	SMG-CMFG	.0126
Pecentral	TH-SFG	.0066
Pecentral	PU-SFG	.0182
Pecentral	CeC-SFG	.0226
Pecentral	BS-SFG	.0066
Insular	IPG-FG	.0008
Insular	ITG-FG	.0304
Insular	LOFG-FG	.0118
Insular	LOFG-ICG	.0412
Insular	LOFG-LOG	.0304
Insular	LG-LOFG	.0304
Insular	MTG-FG	.0304
Insular	IN-ICG	.0234

Insular	IN-LOG	.0304
Insular	IN-PoCG	.0192
Insular	IN-SMG	.0038
Insular	PU-PoCG	.0412
Insular	PU-PCU	.0412
Insular	PA-SMG	.0234
Insular	BS-PaCG	.0424

Supplementary Table 2. SD_STREAM significant edges after TFNBS.

Subgroups	Edges	FWE adjusted P values
Whole	PTR-CMFG	.0078
Whole	PrCG-PTR	.0126
Whole	RACG-MTG	.0454
Whole	RMFG-CMFG	.0126
Whole	RMFG-MTG	.0064
Whole	SFG-MTG	.0008
Whole	SFG-POP	.0126
Whole	SFG-PrCG	.0006
Whole	STG-SFG	.0302
Whole	IN-PoCG	.003
Whole	IN-SFG	.001
Whole	TH-SFG	.003
Whole	PU-LOG	.0454
Whole	PU-PoCG	.0026
Whole	PU-PrCG	.0126
Whole	PU-SFG	.003
Whole	PA-PrCG	.0008
Whole	BS-PrCG	.0008
Whole	BS-SFG	.001
Precentral	MTG-CMFG	.0022
Precentral	PTR-MTG	.033
Precentral	PoCG-CMFG	.0022
Precentral	PrCG-CMFG	.0092
Precentral	RMFG-CMFG	.0036
Precentral	SFG-CMFG	.001
Precentral	SFG-POP	.0016
Precentral	SFG-PrCG	.0006
Precentral	TH-SFG	.0052
Precentral	CA-PrCG	.0138
Precentral	CA-SFG	.0036
Precentral	PU-SFG	.0022
Precentral	PA-PrCG	.0214
Precentral	BS-SFG	.0022
Insular	IPG-FG	.0164
Insular	LOFG-FG	.0228
Insular	IN-PoCG	.0024
Insular	IN-SMG	.0038
Insular	PU-PoCG	.01
Insular	HI-STG	.027

Supplementary Table 3. Significant edges after TFNBS in SD_STREAM and iFOD2.

Groups	Edges
Entire	PTR-CMFG
Entire	RMFG-CMFG
Entire	SFG-MTG
Entire	SFG-POP
Entire	SFG-PrCG
Entire	TH-SFG
Entire	PU-PoCG
Entire	PU-PrCG
Entire	PU-SFG
Entire	PA-PrCG
Precentral	PoCG-CMFG
Precentral	PrCG-CMFG
Precentral	RMFG-CMFG
Precentral	SFG-CMFG
Precentral	SFG-PrCG
Precentral	TH-SFG
Precentral	PU-SFG
Precentral	BS-SFG
Insular	IPG-FG
Insular	LOFG-FG
Insular	IN-PoCG
Insular	IN-SMG
Insular	PU-PoCG
