# Supplementary material for: Network analysis shows decreased ipsilesional structural connectivity in glioma patients
Source: Commun Biol. 2022 Mar 23;5:258. doi: 10.1038/s42003-022-03190-6 (PMC8943189; doi:10.1038/s42003-022-03190-6)
Supplement: Supplementary file 3 — Reporting Summary [file 42003_2022_3190_MOESM3_ESM.pdf]

Corresponding author(s): Lucius Fekonja

Last updated by author(s): 2022.01.24

Reporting Summary

Nature Portfolio wishes to improve the reproducibility of the work that we publish. This form provides structure for consistency and transparency in reporting. For further information on Nature Portfolio policies, see our Editorial Policies and the Editorial Policy Checklist.

Statistics

For all statistical analyses, confirm that the following items are present in the figure legend, table legend, main text, or Methods section.

n/a	Confirmed	
☐	☒	The exact sample size (n) for each experimental group/condition, given as a discrete number and unit of measurement
☐	☒	A statement on whether measurements were taken from distinct samples or whether the same sample was measured repeatedly
☐	☒	The statistical test(s) used AND whether they are one- or two-sided<br>*Only common tests should be described solely by name; describe more complex techniques in the Methods section.*
☐	☒	A description of all covariates tested
☐	☒	A description of any assumptions or corrections, such as tests of normality and adjustment for multiple comparisons
☐	☒	A full description of the statistical parameters including central tendency (e.g. means) or other basic estimates (e.g. regression coefficient) AND variation (e.g. standard deviation) or associated estimates of uncertainty (e.g. confidence intervals)
☐	☒	For null hypothesis testing, the test statistic (e.g. F, t, r) with confidence intervals, effect sizes, degrees of freedom and P value noted<br>*Give P values as exact values whenever suitable.*
☒	☐	For Bayesian analysis, information on the choice of priors and Markov chain Monte Carlo settings
☒	☐	For hierarchical and complex designs, identification of the appropriate level for tests and full reporting of outcomes
☐	☒	Estimates of effect sizes (e.g. Cohen's d, Pearson's r), indicating how they were calculated

Our web collection on statistics for biologists contains articles on many of the points above.

Software and code

Policy information about availability of computer code

Data collection	The datasets and scripts used in this manuscript are available at: https://github.com/CUB-IGL/Network-analyses-reveal-global-and-local-glioma- related-decreases-in-ipsilesional-structural-connect
Data analysis	The datasets and scripts used in this manuscript are available at: https://github.com/CUB-IGL/Network-analyses-reveal-global-and-local-glioma- related-decreases-in-ipsilesional-structural-connect

For manuscripts utilizing custom algorithms or software that are central to the research but not yet described in published literature, software must be made available to editors and reviewers. We strongly encourage code deposition in a community repository (e.g. GitHub). See the Nature Portfolio guidelines for submitting code & software for further information.

Data

Policy information about availability of data

All manuscripts must include a data availability statement. This statement should provide the following information, where applicable:

- Accession codes, unique identifiers, or web links for publicly available datasets
- A description of any restrictions on data availability
- For clinical datasets or third party data, please ensure that the statement adheres to our policy

The datasets and scripts used in this manuscript are available at: https://github.com/CUB-IGL/Network-analyses-reveal-global-and-local-glioma- related-decreases-in-ipsilesional-structural-connect

Field-specific reporting

Please select the one below that is the best fit for your research. If you are not sure, read the appropriate sections before making your selection.

[x] Life sciences [ ] Behavioural & social sciences [ ] Ecological, evolutionary & environmental sciences

For a reference copy of the document with all sections, see nature.com/documents/nr-reporting-summary-flat.pdf

Life sciences study design

All studies must disclose on these points even when the disclosure is negative.

Sample size	We included n=37 left- and right-handed adult patients in this study (15 females, 22 males, the average age was 48.24, SD = 16.47, age range 20-78). Only patients with an initial diagnosis of unilateral WHO grade II, III & IV gliomas (13 WHO grade II, 10 WHO grade III, 14 WHO grade IV) and without a midline shift in structural images were included (Table 1 & Fig. S1). All tumors were infiltrating M1 and/or showing adjacency to the corticospinal tract (CST), either in the left (n=16) or right (n=21) hemisphere. Patients with recurrent tumors, previous radiochemotherapy or non-glial tumors were not considered. The sample size reflects the sample size used in published related studies (cf. introduction/discussion).
Data exclusions	No data were excluded, retrospective analysis
Replication	Codes and scripts are provided
Randomization	Not relevant, we have defined inclusion and exclusion criteria
Blinding	blinding is not relevant due to the design of the study

Reporting for specific materials, systems and methods

We require information from authors about some types of materials, experimental systems and methods used in many studies. Here, indicate whether each material, system or method listed is relevant to your study. If you are not sure if a list item applies to your research, read the appropriate section before selecting a response.

Materials & experimental systems

n/a	Involved in the study	
[x]	[ ]	Antibodies
[x]	[ ]	Eukaryotic cell lines
[x]	[ ]	Palaeontology and archaeology
[x]	[ ]	Animals and other organisms
[ ]	[x]	Human research participants
[x]	[ ]	Clinical data
[x]	[ ]	Dual use research of concern

Methods

n/a	Involved in the study	
[x]	[ ]	ChIP-seq
[x]	[ ]	Flow cytometry
[ ]	[x]	MRI-based neuroimaging

Human research participants

Policy information about studies involving human research participants

Population characteristics	see above
Recruitment	Patients were recruited based on clear inclusion and exclusion criteria
Ethics oversight	Informed consent was obtained after the nature and possible consequences of the study was explained. The study proposal is in accordance with ethical standards of the Declaration of Helsinki and was approved by the Ethics Commission of the Charité University Hospital (#EA1/016/19).

Note that full information on the approval of the study protocol must also be provided in the manuscript.

Magnetic resonance imaging

Experimental design

Design type	dMRI
Design specifications	n/a

Behavioral performance measures | n/a

Acquisition

Imaging type(s) | diffusion

Field strength | 3T

Sequence & imaging parameters | MRI data were acquired on a Siemens Skyra 3T scanner (Erlangen, Germany) equipped with a 32-channel receiver head coil at Charité University Hospital, Berlin, Department of Neuroradiology. These data consisted of a high-resolution contrast enhanced T1-weighted structural scan (TR/TE/TI 2300/2.32/900 ms, 9° flip angle, 256 × 256 matrix, 1 mm isotropic voxels, 192 slices, acquisition time: 5 min) and a single shell dMRI (spin-echo) 2 × 2 × 2 mm3 voxels, 128 × 128 matrix, 60 slices, 3 b0 volumes) image data set, acquired at b = 0 and 1000 s/mm2 with 5 and 30 volumes respectively, for a total acquisition time of 12 minutes. Additionally, T2-weighted and 3D fluid attenuated inversion recovery (FLAIR) and subtraction sequences were performed.

Area of acquisition | whole brain

Diffusion MRI ☒ Used ☐ Not used

Parameters | see above (sequence & imaging parameters)

Preprocessing

Preprocessing software | MRtrix3

Normalization | not normalized, patient specific connectomes were investigated

Normalization template | data were not normalized to standard space

Noise and artifact removal | The preprocessing of dMRI data included the following and was performed within MRtrix3 in order, as described earlier in : denoising, removal of Gibbs ringing artefacts, correction of subject motion, eddy-currents and susceptibility-induced distortions in FSL, and subsequent bias field correction with ANTs N4. Each dMRI data set and processing step was visually inspected for outliers and artifacts. We defined a threshold of scans with more than 10% outlier slices due to excessive motion, however, this was not exceeded in any patient. We upsampled the dMRI data to a 1.3 mm isotropic voxel size before computing FODs to increase anatomical contrast and improve downstream tractography results and statistics. For voxel-wise modelling we used a robust and fully automated and unsupervised method. This method allowed to obtain tissue-specific response functions for white and grey matter and cerebrospinal fluid (CSF) from our data using spherical deconvolution for subsequent use in multi-tissue CSD-based tractography.

Volume censoring | n/a

Statistical modeling & inference

Model type and settings | Statistical analysis was performed by MRtrix3 for connectome group-wise statistics at the edge level using non-parametric permutation testing via the threshold-free network-based statistics (TFNBS) algorithm. The network-based clustering was computed with a default n=5’000 shuffling of data for nonparametric statistical inference. We added the hemispheric tumor position as a covariate to account for hemispheric differences. TFNBS provides multiple hypothesis testing at the level of interconnected subnetworks and controls family-level errors (FWE) in the performance of analyses associated with a particular effect or contrast of interest. TFNBS overcomes some of the limitations of the generic procedure (such as the false discovery rate, FDR), which computes statistical tests and the corresponding p-value independently for each link and considers only the strength of that compound. NBS performs a univariate mass testing procedure to identify those connections that exceed a statistical test threshold and belong to a specific connected component. Finally, a corrected p-value is calculated for each component using the null distribution of the maximum size of the connected component, which is derived empirically using a non-parametric permutation method.

To further characterize the tumor impact on the structural networks, we made use of graph based complex network analyses on the same matrices that were used as input for TFNBS. We used measures of network efficiency to detect aspects of functional integration and segregation. We assessed the global efficiency, a measure of network integration. Global efficiency allows to assess disconnected networks, as paths between disconnected nodes are defined to have infinite length, and correspondingly zero efficiency. Additionally, we measured the local efficiency, a measure of network segregation. Local efficiency reflects the extent of integration between the immediate neighbors of the given node. Furthermore, we computed measures of assortativity, degree, centrality, hierarchy, nodal efficiency, and rich club and small world organization to analyze the vulnerability and resilience of the networks and detect possible abnormalities of network connectivity. These graph theoretical network analyses were performed by GRETNA 2.0.0.

To estimate a rank-based measure of association with neuropsychological assessments, we used the FDR-adjusted Spearman rank coefficient within RStudio 1.3.1093 with R version 3.6.3. The plots were generated with the ggplot2 package.

Furthermore, we performed Pearson correlations to study the relationship between network measures obtained using SD_STREAM- and iFOD2-based tractography.

Effect(s) tested | see above (model type and settings)

Specify type of analysis: ☒ Whole brain ☐ ROI-based ☐ Both

Statistic type for inference (See Eklund et al. 2016)	n/a
Correction	FWE (see above)

Models & analysis

n/a	Involved in the study
☒	☐ Functional and/or effective connectivity
☐	☒ Graph analysis
☐	☒ Multivariate modeling or predictive analysis

Graph analysis	see above (model type & settings)
Multivariate modeling and predictive analysis	see above (model type & settings)
